# “Sometimes I feel like a pharmacist”: identity and medication use among adolescents with juvenile arthritis

**DOI:** 10.1186/s12969-016-0117-1

**Published:** 2016-10-19

**Authors:** J. E. McDonagh, K. L. Shaw, J. Prescott, F. J. Smith, R. Roberts, N. J. Gray

**Affiliations:** 1 Centre for Musculoskeletal Research, Stopford Building, University of Manchester, Oxford Rd, Manchester, England M13 9PT UK; 2 NIHR CLAHRC West Midlands, University of Birmingham, Birmingham, UK; 3 School of Education and Psychology, University of Bolton, Bolton, England UK; 4 UCL School of Pharmacy, London, England UK; 5 Pharmacy Research UK, London, England UK; 6 Green Line Consulting Limited, Manchester, England UK

**Keywords:** Young people, Rheumatology, Identity, Medication, Qualitative

## Abstract

**Background:**

Taking medicines as intended is difficult for everybody, but young people going through adolescence have greater problems than adults and younger children. One of the most important things that happen during the teenage years is the development of individual identities, which might not remain constant during this time and can be affected deeply by the diagnosis of a long-term condition. The aim of this study was to examine the relationships between identity and medication use among young people with juvenile arthritis.

**Methods:**

A prospective qualitative study was undertaken to collect private online ‘blog’ style data from young people (aged 11–19 years) with juvenile arthritis, and their parents, to examine their views about their condition, identity, medication and use of health services. Participants were identified from a large paediatric hospital in the UK.

**Results:**

Young people (*n* = 21) with a median age 14 years (range 11–17 years) posted a median (range) of 8 (1–36) blogs and parents (*n* = 6) posted 4 (1–12) blogs. Young people gave a strong sense of both private and public identity that was intertwined with their arthritis and treatment. It was evident that young people’s self-care was intrinsically linked to their attempts to maintain a sense of individually and socially constructed definitions of normality. The act of taking medication, and the consequences (positive or negative) of that act, had an impact both personally and socially.

**Conclusions:**

Young people with juvenile arthritis reflect on their medication as a factor affecting their perception of themselves. Acknowledging the roles of both personal and social identity will be important in any strategies to support optimal medication use. This includes an understanding of the identity transformations that young people can experience and how decision-making may be affected by their attempts to retain pre-diagnosis identities and/or develop new social identities.

## Background

Age and developmentally appropriate services, with particular reference to adolescents, have been reported nationally and internationally as a key mechanism to improve health outcomes for young people [1]. This has been recognised in rheumatology for over a decade [2]. Unfortunately, while there have been some encouraging advances in youth-friendly care [3], many young people remain inadequately prepared to manage their condition in adulthood [4, 5]. Difficulties around medication are particularly evident, with suboptimal adherence reported in several studies [5–7]. The problems faced are many and complex, including negotiating the shift from parental- to self-management, and integrating medicine-taking within demanding daily routines [5–7]. Young people can also demonstrate a lack of basic knowledge about their medications [5, 8, 9] and report significant concerns about side-effects and drug-dependency [5].

Helping young people to manage their medication and concerns is therefore a critical aspect of self-care. Not only is this congruent with adolescent development [10], but also a predictor of successful transition to adult care [11–13]. While there is evidence, however, that young people want to assume self-care responsibilities [6, 14, 15], and that some rheumatology professionals encourage them to do so [11, 12], effective self-management training is not routinely provided [16, 17]. The implications of this include adverse health outcomes, reduced quality of life and lost healthcare resources [7, 18].

Unfortunately, little is known about the optimal approach for self-management during adolescence. A recent synthesis of qualitative research shows that while medication management is important to young people, arthritis and its treatment can profoundly affect their sense of self [6]. Identity formation is one of the key developmental tasks of adolescence [10]. This includes both personal (or private) identity; “a definition and evaluation of oneself in terms of idiosyncratic personal attributes or one’s relationships with specific people” and social (or public) identity; “a definition and evaluation of oneself in terms of shared attributes that define membership of the specific group one belongs to” [19]. However, while the experience of living with arthritis is well documented [6], there is scarce information about the relationship between identity and medication use. Understanding these relationships will be potentially helpful for health professionals when providing support to young people with long term conditions.

## Methods

The aim of this study was therefore to examine the relationships between identity and medication use among young people with juvenile arthritis.

A prospective study (the ‘Arthriting’ project) [20, 21] was undertaken to collect private online ‘blog’ style data, employing principles of qualitative methodology from young people with arthritis, and their parents, to examine their views about their condition, identity, medication.

### Participants and setting

Young people and their parents were identified from the rheumatology database in a large UK paediatric hospital. Eligibility criteria for young people required that they had confirmed juvenile arthritis, were aged 11–19 years, and had no cognitive impairment that would prevent their ability to provide data. Diagnoses of Juvenile arthritis included juvenile idiopathic arthritis as defined by ILAR criteria [22] in addition to other inflammatory arthritides prevalent during adolescence eg lupus or inflammatory bowel disease associated arthritis.

### Procedure

A detailed description of the methodology of this study was already been published [ref]. In summary, young people (and their parents) were invited to participate during routine clinics (*n* = 70) and by letter (*n* = 107). Consenting participants were given an information pack about the ‘Arthriting’ website and a personal login code. To maximise relevance and uptake, development of the website was undertaken in partnership with young people’s user groups and included stringent security processes to ensure data protection and participant safety (e.g. password protected logins, secure firewalls, regular moderation). The website was also functional on Smartphones, recognising that many young people (and parents) access the Internet this way. Once registered, participants were asked to choose a nickname and password for future logins. Once logged in, participants were asked to choose from a number of blog categories [21] and enter their thoughts. This list had been generated by the research team to give young people ideas for the topics and types of messages that they might post (eg, thoughts about identity, the arthritis condition, medication, and the use of health services). These “blog categories” were developed and refined in discussion with the young people. They were also able to add an emoticon to reflect their mood at that time. The choice of categories, frequency of access and lengths of engagement was determined by participants, who could also edit blogs at any time during the 2-month active period. On completion of the 2-month period, participants who contributed at least one blog were sent a thank you letter and a £50 online shopping voucher.

### Analysis

Blog entries for each participant were uploaded into QSR NVivo 10 (2012) as separate files and tagged with their nickname, date of posting, and emoticon. With permission, basic (non-identifiable) demographic and relevant clinical data were also added (i.e. age; sex; ethnic group; age at diagnosis, and number of medications). The data were analysed thematically using a ‘middle-order’ approach [23] within a framework of ‘directed content analysis’ [24]. This involved rigorous procedures to enable themes and categories to be generated from the data through processes of induction (i.e. moving from the data towards generalizations, hypotheses, or theory). This approach was possible because the blog activity was designed to generate data in relation to specific research objectives and thus provided a clear source of categories with which to organise participants’ responses, whilst allowing other themes to emerge. Data was analysed by six experienced researchers. Primary attention was directed at identifying broad categories of data, which were used to develop an agreed codebook. Researchers then worked in pairs and were assigned a third of blog entries to analyse. This involved independent specific line-by-line categorisation, followed by paired discussions to establish the level of agreement and resolve discordance. Whole group consensus on the coding was established during several teleconferences.

## Results

### Participant characteristics

In total, 36 (20.3 %) young people and 6 parents consented to participate (Table 1). Of these, 25 (14.1 %) registered to participate and 21 (11.9 %) completed at least one blog. Of those young people who blogged, median age at recruitment was 14 years, most were female (*n* = 17, 81.0 %), white (*n* = 17, 81.0 %), and had Juvenile Idiopathic Arthritis (*n* = 19, 90.5 %) with a median age at diagnosis of 2.5 years. The median number of medicines being taken was 2 (1–6).

**Table 1:** Characteristics of young people who contributed at least one blog

Characteristic	Values	Number of participants
Age at recruitment	11–15	18
	16–19	3
Gender	Female	17
	Male	4
Ethnic group	White	17
	Non-white	4
Age at diagnosis	<11 years	13
	11+ years	8
Time since diagnosis	1–5 years	8
	6–10 years	4
	11+ years	8
	Not known	1
Type of juvenile arthritis	Juvenile Idiopathic Arthritis JIA	19
	Other	2

Young people posted a median of 8 (1–36) blogs and parents posted a median of 4 (1–12) blogs. The unedited blogs are presented here as written by the participants.

### Impact during adolescence of arthritis on personal identity

It was evident that adolescence posed distinct challenges for young people and their parents (Table 2a); young people had a sense of what was ‘normal’ for them based on past experiences. This varied depending on their age at disease onset (Table 2b). Those with an early onset (during infancy and early childhood) described how arthritis was a ‘normal’ part of their self, and how medication use was a routine part of life. Those diagnosed in later childhood or adolescence, however, described diagnosis as a life-changing event. Both parents and young people frequently contrasted life *before* and *after* diagnosis, describing how arthritis had profoundly affected young people’s views of the world and how they acted within it. The response of many young people was to try and retain as much of their pre-illness self as possible. Unfortunately, the success of this was variable, leaving some young people frustrated and disappointed. This was further compounded by the remitting/relapsing nature of arthritis and its treatment which could alter their ‘usual’ self on a temporary or episodic basis (Table 2c).

**Table 2:** Blog reflections upon arthritis and personal identity

*a. Early and late diagnosis:*
* I was diagnosed when I was tiny so I don’t know any different. in a way I think it would be weird to not have it but that’s probably since I have grown up with it being part of my life, but not letting it rule it. [Female, 16 years old]*
* I used to do a lot of running, and when I was diagnosed I was unable to do it. I do at times miss it, and I do feel that people saw me differently. I only say this because I was known as a runner, and when I was unable to run anymore I was almost isolated from the rest of my peers. [Female, 16 years old]*
* My Daughter has never really known any different in her life as she was diagnosed with arthritis at such an early age, I suppose that has helped in a way really as she just gets on with things and knows her limitations and when to stop. As she has approached her teenager years her arthritis does get her down and when her friends are doing something that she knows will tire her out or that she just could not do she does go quite moody and frustrated. [Parent]*
*b. Effects upon forming identities:*
*Does arthritis affect my identity? I don’t really think it does me still can do what every other kid can do! I still do all my hobbies* e.g. *Judo, swimming and dance. My life is pretty ordinary if you take away all the injection and blood tests…. [Male, 11 years old]*
*I am a very sociable person when I am happy on a good day, however if I am caught on a bad day with regards to my arthritis and my mood, then I can be the complete opposite. [Female, 16 years old]*
*c. Impact in adolescence:*
*I am fifteen years of age and have had arthritis most of my life, I was only about 2 years of age when my parents were told that I had the disease, I suppose when I was younger I sort of just got on with it, but as a teenager it can effect me in a variety of ways. [Female, 15 years old]*
*We spent the first 10 or 11 years hoping that she’d grow out of it at puberty only for her to suffer more around that time. [Parent]*
*Our daughter takes her methetraxate on Friday evenings. By Saturday afternoon she feels sick and is quite down. This continues until around Tuesday where she perks up and becomes her old self. Still, is this the effect if the meds or her mental state or just teenage angst? [parent]*
*d. A positive force:*
*but im glad that it happened in away because i made a better group of friends and the(y) did not understand at first, but they were willing to and now they are my greatest friends. [Male, 15 years old]*
*If i could change myself eg: not having arthritus i dont know if i would because it has made me a stronger person… and i have faced some of my fears like operations and needles and im proud of my self for that…. [Female, 13 years old]*
*e. Facing the future:*
*Today I am not feeling too good. My arthritis is really playing up on me today. I feel really down and tired. Mainly grumpy and unsociable. It’s on days like this when I ask myself, ‘How am I going to cope with working in the working world when I can’t even cope with just going to college?’ [Female, 16 years old]*
*“as a mother, at the moment, I’m feeling very worry about what the future holds for my son regarding his arthritis. NextSeptember he is moving tosecondaryschool, thatnormallywill be an scary time for any mother but when your child has a cronicalillnessthe idea of him growing without knowing how his condition is going to affect him is veryscary. What happens if he doesn’t take the medication? I just wish it was more information for parents on how to deal with all this worries! I also feel afraid about the moment when he leaves the doctors in the children hospital. Will I be able to trust any other doctor with him?” [Parent]*

Young people strived to engage in behaviours that were defined as developmental ly and culturally normal. While this could be detrimental to their physical health (e.g. exacerbating symptoms), young people were very mindful of how they appeared to others and lack of participation was felt to have significant consequences for young people’s sense of belonging (Table 2b,c). Interestingly, some young people also described their arthritis and/or treatment as a positive force in their lives (Table 2d).

Commensurate with their increasing cognitive abilities, young people were beginning to consider their futures. However, forming and consolidating an identity that incorporated the future self could be extremely challenging, especially given the remitting/relapsing nature of arthritis (Table 2e).

### Medication, medicine management, and person identity

For some young people, medication affected their sense of self (Table 3a). The value of medication was intrinsically linked to its potential to protect or threaten the young person’s self-identity. Thus, while some young people described their health regimens as onerous and unwelcome, they felt that the real and anticipated benefits outweighed the level of disruption (Table 3b). Many young people acknowledged their need for the medication, seeing it as important if they were to stay well and for longer term disease outcomes. Thus, young people were often faced with a paradox. Effective disease management could help them maintain a sense of self by minimising symptoms, the visibility of their condition and enabling them to participate in identity-confirming activities. Yet, at the same time, medication (and other aspects of treatment) were both a public and private reminder of their illness. Visible side-effects of medication, in particular, could disrupt a sense of self (Table 3c).

**Table 3:** Blog reflections upon medication (including medication management) and personal identity

a. Effect of medication upon identity
*It hasn’t affected the way i value myself; i still think the same of myself that i used to. My illness doesn’t really affect me apart from when i have to take my medication other than that i can’t feel it. I don’t think any less of myself because why should i???? Just because i have an illness...... I think that is just stupid. I am perfectly normal and i think that i am the same person i was before i got diagnosed. I think no less of myself. [Female, 13 years old]*
b. Side effects
*The medication I have can give me a weird reaction, and it makes me put weight on more easily, so I am used to being bullied by people calling me ‘chubby cheeks’ and other mean stuff, but my friends have always helped with bullies, by making me feel better, and getting me through harsh comments. [Female, 13 years old]*
*The doctors want me to go on another medicine as well as although the embrel does make my joints feel better im still in alot of pain and can’t walk far, however i feel a bit nervous to go on it as it can have alot of side affects just like any medicine can. Therefore i have to think about what benefits me more and i know that this medicine will be helpful. [Female, 15 years old]*
*Had to give the twice weekly injection - never a very nice time, it takes quite a while for this procedure as she is always very nervous that it will hurt, even though she has had more injections in her lifethan I have had in the whole of mine giving her her injection is never easy.[parent]*
c. *Weighing up the benefits vs the disruption*
*To be honest, I HATE taking medication everyday. I know that in the long run, it could help me with my symptoms, however it isn’t nice having to take them all the same. (Female, 16 years old)*
*Taking medication is not the easiest thing in the world, it gets quite annoying knowing when to take it, but i don’t mind as long as it makes me better. [Male, 15 years old]*
*‘…I know without it I could become ill again and I don’t want to end up in hospital again.’ [Female, 13 years old]*
d. Medication management
*To be honest, there aren’t that many positives when it comes to taking medication. I know that I have said this many times, but I feel that the main positive that has come from taking medication is having a sense of independence. I absolutely hate taking medication, but I cannot deny that it has given me an insight of taking responsibility for things. (Female, 16 years old)*
*I kind of enjoy being in charge of my medication because sometimes I feel like a pharmacist??? [Female, 13 years old]*
*Now I’m kinda like an expert on medication i know all the doses and names of my medications. [Female, 13 years old]*
*Do i get enough help from my friends and family??? Yes i do everyone helps me in every way they can although i don’t need a lot of help. My friends don’t really need to help however they do sometimes ask have you had your medication? I am in charge of my medication therefore i am responsible for it. My mother helps by getting my prescription and collecting the medication from the chemist as i am not aloud because i am to young. [Female, 13 years old]*
*I feel really pleased that my daughter has taken full responsibility for taking her medication, there have only been a few times when she has forgotten to take it [parent}*
*The thought of our son been in control of the medication is a very frightening thought. As parents we know that the moment will have to arrive so he becomes responsible of his well being and independent but never the less still a worry because when you are in control your mind is at rest that you are doing it right but when you have to trustsome else with the medication becomes another thing for your head to worry about!!! [parent]*

Young people were increasingly responsible for their medicine management, and while it was often seen as onerous, it could also be empowering (Table 3c). Young people described making different self-care choices to manage their social identities within the demands of different situations. Life within the family was rarely seen to threaten the young person’s sense of normality, and thus young people and parents often worked constructively to negotiate and support greater independence in using medications. However supporting their child’s developing autonomy was not always easy for the parents (Table 3d)

### Arthritis, medication use and public identity

Although many young people were developing a personal identity that incorporated arthritis and/or its management, it was clear that young people also wanted to create and maintain a public identity that adhered to social definitions of normality. Some young people therefore described how arthritis and its treatments led to experiences of normality which differed significantly to those of others, particularly their peers. This was compounded by the limited representation of juvenile arthritis in society or public understanding (Table 4a). Disclosure, of any type, often meant that young people with arthritis needed to ‘explain themselves’ and this was often a source of frustration, especially when explanations were not fully accepted or understood.

**Table 4:** Blog reflections upon arthritis, medication and public identity

*a. Societal Attitudes*
*People think that arthritis is for old people, which is not true quite sad to be honest when they compare you to old people [Female, 15 years old]*
*I often find that explaining to people what arthritis is and why you go to hospitals alot, it gets tiring and annoying especially when they end it with comments like ‘my nan has that’ or whatever. Yes your nan may have it but others can get it too! I am however lucky to have my boyfriend as he too is familiar with hospitals and i can totally rely on him to carry me when im in pain.. Result! :)[Female, 15 years old]*
*b. Normality through limiting disclosure*
*It doesn’t really affect me because i don’t really tell people about my illness because i don’t want them to treat me differently. I think that people do treat you differently when they find out because they don’t no about the illness. I don’t like that because i prefer it when people take no notice and they shouldn’t because i may have an illness but that doesn’t make me any different from everyone else. However my friends take no notice of my illness and are just glad that i am well now. [Female, 13 years old]*
*applied for college last night and there was a section about illnesses, had to describe my arthritis, sometimes school does not understand, like on Tuesday we had assembly and the chairs in the rows are really close together, it hurts my legs to keep them in that position for all that time, mom keeps on telling me to tell them but I feel awkard about it, hopefully college will be different. [Female, 15 years old]*
*c. Social Networks*
*Cannot talk to my friends about it [methotrexate] as they would not understand so me and mom discuss it and she assures me all will be ok. [Female, 15 years old]*
*I have a lot of caring friends who help with many things in and out of school. They all understand everything i have to put up with and i never have to worry about going out and not being able to walk after a period of time as i know that if i need to rest they will sit with me until i’m ready to get going again. My friends never hesitate to help me out when i’m struggling, e.g if i’m walking home from school and i have lots of bags to carry, they always offer to give me a hand and help me out. [Female, 15 years old]*
*ive got a confirmation meeting tonight and i think its lovely going to church and finish off my sacrament. Its lovely what ever youve got like arthritus, asma or anything else its lovely to know your apart of something and its lovely i cant wait! [Female, 13 years old]*
*I do get very fustrated with it all because of how active i used to be and now i struggle to do most things, and I know teachers and friends understand but i dont think they realise the constantness of it all, that when i wake up i need to check if i can move properly today and i dont think my parents realise either because its always there so theyve just learnt to ignore it because its constantly there and i do accept that ive got it [Female, 15 years old]*

A recurrent theme in the blogs was the need to minimise difference in order to form and maintain a positive social identity. A common strategy used by young people was to conceal or minimise the significance of their arthritis by reducing the visibility of their condition, including self-management activities, and limiting disclosure (Table 4b). Yet, paradoxically, invisibility was itself a problem because others did not always appreciate the impact of arthritis. Young people therefore have to make decisions about disclosure, balancing its potential benefits and risks.

Young people belonged to varied social groups and presented different images of themselves depending on the context (Table 4c). While arthritis could be highly disruptive in some social settings, it was perceived as less relevant in others. Young people therefore described making different self-care choices to manage their social identities within the demands of different situations. Close friends and romantic partners were often brought into the ‘circle of trust’. These relationships appeared to support - rather than threaten - young people’s social identity, given their defining features of reciprocal self-disclosure, support and mutual interest.

## Discussion

Young people gave a strong sense of both private and public identity that was intertwined with their arthritis and medication use. The overarching theme was one of ‘normality’ which echoes the findings of a recent review of qualitative studies that describe the experiences of children and adolescents living with juvenile idiopathic arthritis [6, 25], as well as those observed in other conditions (e.g. liver transplantation [26] haemoglobinopathies [27], Cystic Fibrosis [28]). However, by focusing on identity and medication use, this study adds to the current knowledge base by revealing how the desire for normality influences medication use. Specifically, it shows that young people judge the value of their medicines against their potential to support or disrupt their perceptions of what is normal for them, and their attempts to ‘fit in’ with socially constructed views of normality. From this stance, medicine management therefore needs to start with an understanding of how young people see themselves, how they think others see them, and their goals and aspirations.

The relationship between arthritis, treatment and identity appeared particularly complex for young people diagnosed in later childhood and adolescence, who described their attempts to reconcile life before and after onset. Parallels can be drawn with the sociological concepts of ‘biographical disruption’ [29] and ‘loss of self’ [30] which describe how chronic conditions can alter the ‘taken-for-granted’ features of life and force individuals to reappraise the meaning of their lives. Although these concepts derive from adult studies, the responses of many young people were comparable, in that they tried to preserve their pre-illness identity (sometimes by concealing their condition and treatment), with some also showing evidence of developing an altered identity that incorporated arthritis as an accepted (and sometimes positive) component of life. Moreover, Grinyer [31], in her work with young people with cancer, suggests that the potential for biographical disruption is exacerbated in adolescents, given that they are at a transitional stage of life where identity is particularly fragile and developmental goals include increased independence. Others additionally suggest that the development of identity is further complicated at this stage of life by the ‘inherent tensions’ between the concept of youth (typically portrayed as a time of health, beauty, strength etc.) and illness/impairment [32, 33]. This is not to say that identity formation is necessarily easier for young people diagnosed in early childhood. Regardless of onset age, adolescence raised additional challenges for young people, including consideration of their place in the world and the future. Thus, compared to adults, whose illness requires them to re-construct an already established identity, the young people in this study were presented with the arguably more complex challenge of creating and consolidating an identity in the context of a condition that may fluctuate, have uncertain outcomes and is typically represented as a disease of older adulthood. As such, the findings lend support to the conclusions of Williams et al. [28] who, in the context of cystic fibrosis, suggest that the task faced by young people is to continually revise their biographies “in anticipation of future illness trajectory and life course” (p.1443).

Effective medicine management strategies are likely to require a meaningful exploration of young people’s support needs across a range of social contexts. Newbould et al. [34] caution against judging a patient’s potential to manage their condition based on clinic behaviour. They found that while young people with asthma and diabetes played a considerable role in managing their medicines at home, they often adopted a passive role in clinic consultations. It is important, therefore, to provide opportunities for young people to make care plans that acknowledge their desires for self- and socially- referenced normality and which include reference to their goals and aspirations. Given the loss of self that many young people experience, it will also be important to highlight the gains that can be experienced through self-care activities, as described in this study (i.e. sense of personal growth and mastery). Young people may also feel more motivated if they have a greater understanding of how self-care activities can facilitate participation in their valued activities, and how symptoms and side-effects that threaten identity could be minimized or managed. This is important as the perceived burden of medication has been shown to predict impaired health related quality of life in young people with JIA [35].

There remains much to be done to increase the social representation of arthritis in young people. As in other studies [6, 36], young people and parents were often concerned about the lack of social understanding, particularly at school and work. This is of relevance in view of the reported concerns regarding vocational outcomes for young people with long term health conditions [37], and the increasing interest in rheumatology research in this area [38, 39].

The findings presented here underscore the need to provide young people with permissive environments in which to explore medication use in the context of their everyday lives. This reflects the central tenets of self-categorisation theory [40] that regards young people as having a variety of social identities which become salient in different contexts. In this context, medication use (and other aspects of self-care, including disclosure) is seen as a choice, but one that is subject to the context of the specific social situation. Thus, effective medicine management strategies are likely to require a meaningful exploration of young people’s support needs across a range of social contexts. Interviewing tools such as HEADSSSS are likely to support such strategies [41, 42] although such psychosocial screening is not yet standard practice [17, 43]. Implications for clinical practice are detailed in Table 5. This data has since informed a further study of pharmacists’ perceived role in the care of young people with rheumatic disease using JIA as an exemplar disease [44], the paper of which has been submitted to the Journal of adolescent health and is in the revision stage. As a result of the latter study, a trial of a pharmacy-led intervention to improve medicine management skills, patient reported outcomes and reduce healthcare resource use through improved engagement in health care is currently in the development phase.

**Table 5:** Implications for practice and further study

• Provision of permissive environments in which to explore medication use with young people in the context of their everyday lives.	
• Effective medicine management strategies which explore young people’s support needs across a range of social contexts	
• Provision of opportunities for young people to make care plans that acknowledge their desires for self- and socially- referenced normality and which include reference to their goals and aspirations.	
• Enhance young people’s understanding of how self-care activities can facilitate participation in their valued activities and how symptoms and side-effects that threaten identity could be minimised or managed.	
• Enhance young people’s understanding of how symptoms and side-effects that threaten identity could be minimised or managed.	
• Skills training for young people which will enable them to negotiate the social situations that they are likely to encounter eg opportunities to develop and practice positive disclosure skills	

### Limitations

The novel way of engaging with young people and parents in this study has resulted in rich in-depth data about the relationships between arthritis, identity and medication use. However, several caveats should be borne in mind when interpreting the findings. Firstly, the response rate was low and the sample was self-selected, possibly reflecting those who felt competent with online activities and had reasonable literacy skill. The requirement for access to a smartphone or computer may also have excluded young people of lower socioeconomic status. Responses may have been improved if we had offered a choice of data collection methods (e.g. interviews, focus groups) and may have supported wider participation, including those with no/limited confidential access to the Internet. Given the relatively small number of participants, there are limits to the inferences that can be made. This is discussed further in the methodology paper [22]. However, there were a number of consistent and identifiable themes and while not all participants gave evidence of every theme, common patterns of meaning were discerned and have been presented. A wider age range and longer follow-up may also have provided greater insight into how identity changes over time, especially given that much identity development also occurs in late adolescence and sometimes not until young adulthood [45].

The low response rate is in itself of interest in view of the increasing interest in the use of ehealth and mhealth interventions with this age group in health care [46]. Young people were however involved from the outset including in the design of the blogging site to try and enhance both recruitment and retention. Further study of the potential of such technologies in both research and clinical settings is awaited.

## Conclusion

The use of a novel methodology to empower young people to express their feelings about living with juvenile arthritis has provided a naturalistic description of their concerns and triumphs. Young people with juvenile arthritis reflect on their medication as a factor affecting their perception of themselves. The physical act of taking medication, and the consequences (positive or negative) of that act, have an impact both personally and socially. Family and friends may reinforce these associations with well-intentioned reminders. The exploration of the impact of arthritis and medicine-taking on identity, and the young person’s feeling of normality, may offer healthcare providers further ways to achieve concordance and thus to optimise adherence.
